# Resistance Training with Single vs. Multi-joint Exercises at Equal Total Load Volume: Effects on Body Composition, Cardiorespiratory Fitness, and Muscle Strength

**DOI:** 10.3389/fphys.2017.01105

**Published:** 2017-12-22

**Authors:** Antonio Paoli, Paulo Gentil, Tatiana Moro, Giuseppe Marcolin, Antonino Bianco

**Affiliations:** ^1^Department of Biomedical Sciences, University of Padova, Padova, Italy; ^2^Faculdade de Educação Física e Dança, Universidade Federal de Goiás, Goiania, Brazil; ^3^Department of Nutrition and Metabolism, University of Texas Medical Branch, Galveston, TX, United States; ^4^Sealy Center on Aging, University of Texas Medical Branch, Galveston, TX, United States; ^5^Sport and Exercise Sciences Research Unit, University of Palermo, Palermo, Italy

**Keywords:** aerobic capacity, strength training, resistance exercise, fat loss, muscle strength

## Abstract

The present study aimed to compare the effects of equal-volume resistance training performed with single-joint (SJ) or multi-joint exercises (MJ) on VO_2_max, muscle strength and body composition in physically active males. Thirty-six participants were divided in two groups: SJ group (*n* = 18, 182.1 ± 5.2, 80.03 ± 2.78 kg, 23.5 ± 2.7 years) exercised with only SJ exercises (e.g., dumbbell fly, knee extension, etc.) and MJ group (*n* = 18, 185.3 ± 3.6 cm, 80.69 ± 2.98 kg, 25.5 ± 3.8 years) with only MJ exercises (e.g., bench press, squat, etc.). The total work volume (repetitions × sets × load) was equated between groups. Training was performed three times a week for 8 weeks. Before and after the training period, participants were tested for VO_2_max, body composition, 1 RM on the bench press, knee extension and squat. Analysis of covariance (ANCOVA) was used to compare post training values between groups, using baseline values as covariates. According to the results, both groups decreased body fat and increased fat free mass with no difference between them. Whilst both groups significantly increased cardiorespiratory fitness and maximal strength, the improvements in MJ group were higher than for SJ in VO_2_max (5.1 and 12.5% for SJ and MJ), bench press 1 RM (8.1 and 10.9% for SJ and MJ), knee extension 1 RM (12.4 and 18.9% for SJ and MJ) and squat 1 RM (8.3 and 13.8% for SJ and MJ). In conclusion, when total work volume was equated, RT programs involving MJ exercises appear to be more efficient for improving muscle strength and maximal oxygen consumption than programs involving SJ exercises, but no differences were found for body composition.

## Introduction

Resistance training (RT) is known to have positive effects on health (Kraemer et al., 2002a; Steele et al., 2017b), weight control (Paoli et al., 2014), and performance (Deschenes and Kraemer, 2002; Kraemer et al., 2002b). Designing RT programs is difficult due to the great number of variables involved (Tan, 1999; Paoli and Bianco, 2012), underscoring the proposition that RT should be investigated more thoroughly and rigorously by taking into account these different variables (Paoli, 2012; Paoli and Bianco, 2012; Gentil et al., 2017a). One of the many variables that coaches and researchers face when designing RT programs is exercise selection (Gentil et al., 2017c). Resistance exercises can be classified according many different criteria, considering the number of joints involved they can be classified as multi-joint (MJ) or single-joint (SJ) exercises. Although MJ and SJ have many differences, there is no precise guideline indicating which one is more suitable for different outcomes. Most popular recommendations postulate that RT sessions should involve 8 to 10 exercises performed in multiple sets with both single (SJ) and multi joint (MJ) exercises (ACSM, 2009; Garber et al., 2011).

Previous studies have compared the effects of adding SJ exercises to a MJ exercise program and found no benefit regarding elbow flexors strength and size, either in untrained (Gentil et al., 2013), or trained subjects (de Franca et al., 2015). The only known study to directly compare SJ and MJ exercises was performed by Gentil et al. (2015), who found no difference in elbow flexors size and strength between a group performing only MJ (lat pull down) or a group performing only SJ exercise (biceps curl). Although the results are initially interesting, the protocols did not equate training volume between groups. This might be an important caveat, since the benefits of MJ exercise is purported to be related to higher training volume, reflected in higher hormonal and metabolic responses compared to those from SJ exercises (ACSM, 2009). Therefore, the lack of differential findings concerning elbow flexors strength and size could have been influenced by total work, as MJ exercises usually allow more weight to be lifted than SJ exercises.

The debate around MJ and SJ exercises has been centered on muscle activation, strength and hypertrophy (Gentil et al., 2017c); however, more recently, the benefits of RT on metabolism, weight loss and aerobic capacity have been increasingly recognized (Paoli et al., 2010, 2013; Warner et al., 2010; Fisher and Steele, 2014; Verdijk et al., 2016; Barbalho et al., 2017; Muñoz-Martínez et al., 2017), as is the case for combining endurance training with either explosive or heavy strength training to improve running performance (Rønnestad and Mujika, 2014). Therefore, studying the effects of different exercise types in other, non-traditional outcomes may be useful in alternate areas, such as athletic training and metabolic disease treatments. In light of this, the purpose of the present study is to compare the effects of RT performed with MJ or SJ exercises with equivalent work volume on aerobic capacity, body composition and muscle strength of young active males.

## Materials and methods

### Study participants

Participants were invited though records kept from previous studies conducted by the research group. Thirty-six young males (28 ± 4.5 years, 174 ± 3 cm, and 80 ± 3 kg) accepted the invitation to participate in the study. The participants were amateur soccer players with no previous experience in RT. All were screened by a Sport Physician for the presence of diseases or conditions that could place them at increased risk for an adverse event due to the study protocol. The experiment lasted 12 weeks and was performed during the pre-season. Subjects underwent 4 weeks of familiarization during which they performed a training protocol involving bench press, lat pulldown, military press, barbell biceps curl, push down, knee extension, leg press, and leg curl. All exercises were performed with 3 sets of 15 repetitions at 60% of 1 RM and 1 min of recovery between sets. After the familiarization period, the participants were randomly assigned to one of the experimental groups and performed 8 weeks of training. The volunteers were instructed to not change their nutritional habits during the study period and were constantly monitored to detect the occurrence of any major alteration (i.e., becoming a vegetarian, restricting calories, taking nutritional supplements, or ergogenic aids, etc.). Prior to acceptance for study participation, the volunteers read and signed an informed consent form, which contained a detailed explanation about the study protocol. The study conformed to standards for the use of human subjects in research as outlined in the current Declaration of Helsinki and was approved by the Ethical Board of the Department of Anatomy and Physiology of the University of Padova (DAF-U 12-3).

### Body composition

Anthropometry was obtained with participants barefoot and wearing light clothing. Weight was measured to the nearest 100 g with a calibrated electronic scale. Height was assessed to the nearest 0.1 cm with a wall-mounted stadiometer.

Body composition was assessed by Dual Energy X-ray Absorptiometry (DEXA) with fan-beam technology (Hologic QDR 4500W, Inc.). The participants were instructed to hydrate normally, to avoid the ingestion of any substance or food that could influence body fluid content (i.e., caffeine, creatine, alcohol, tea, and foods with high sodium content) and to not perform any form of physical activity during the day before the test. Before the scan, participants were asked to remove any metal objects, such as jewelry, that could attenuate the X-ray beam. Participants were positioned on the DEXA table according to the protocol recommended by the supplier, in which the subject laid supine and motionless with arms alongside. Before the scanning session, the equipment was calibrated according to the standard procedures supplied by the manufacturer. All scans were acquired and analyzed by the same experienced operator, adhering to the guidelines provided by the manufacturer.

### One repetition maximum (1 RM) test

Maximal strength was determined by assessing 1 RM for the bench press, knee extension and squat (Technogym SpA, Gambettola, Italy) 1 day after the body composition test. The 1 RM test for the squat and bench press were performed using barbells and weight plates starting at 1 kg. Knee extension was performed using a standard resistance training machine. Tests were preceded by warm up (10 min at a comfortable speed on a treadmill) and a specific warm up of 5 repetitions with a weight they could normally lift 10 times. The weight was gradually increased until the participant could only perform one repetition. Each subject had a maximum of five attempts to achieve his 1 RM load, and the rest interval between attempts was 5 min (Bianco et al., 2015). Subjects received verbal encouragement throughout the test, and the same investigator performed all testing procedures. The test-retest reliability in our laboratory for 1 RM varies from 0.92 to 0.97 (ICC) for the exercises tested.

### Aerobic power

Peak oxygen consumption (VO_2_max) was measured during a maximal incremental test using a cycle ergometer (Ergoselect 200, Ergoline GmbH, Bitz (Baden-Württemberg, Germany). Before the test, the volunteers rested quietly for 5 min, then the test started with 3 min of cycling at 20 W; the work-rate was increased by 1 W every 3 s (20 W/min) until the participant was unable to continue pedaling despite encouragement. The participants cycled at a self-selected pace that remained constant throughout the test (70–90 rpm). Heart rate was continuously monitored with electrocardiography. During the test, subjects breathed trough a face mask for a breath-by-breath analysis of oxygen uptake (VO_2_) and carbon dioxide production (VCO2) using an Ergocard® ergospirometer (Pacific Medical Systems, Hong Kong, S.A.R) and a “pitot tube” pneumotacograph equipped with standard gas analyzers. The system was calibrated before each measure using calibration syringes and precision oxygen and carbon dioxide gas mixtures. Subjects were requested to abstain from caffeine or alcohol consumption for 24 h prior to the measurement. Bland-Altman plots and comparison of the test-retest measurements performed in our laboratory confirmed good reproducibility of the measurements for VO_2_ (ICC >0.9 with *p* < 0.05). The aerobic tests were performed 2 days after 1RM tests which, for the post-training testing, occurred 3–5 days after the last training session.

### Training program

The training period lasted 8 weeks and involved 3 weekly RT sessions. Participants were divided in two groups: SJ group (*n* = 18) exercised with only SJ exercises (e.g., dumbbell fly, knee flexion, etc.), MJ group (*n* = 18) with only MJ exercises (e.g., bench press, deadlift, etc.), as shown in Table 1. Participants were encouraged to perform every exercise to momentary muscle failure as previously defined by Steele et al. (2017a), and loads were adjusted set to set to keep repetitions inside the specified range. MJ exercises were performed with 6–8 maximum repetitions (RM). However, because the loads used during SJ were lower than loads used during MJ exercises, it was necessary to increase the number of repetitions for SJ exercises. Work volume matching was performed individually during the familiarization period. In general, to match the work performed during 6–8 RM in MJ exercises, it was necessary to perform 12–18 RM in the SJ exercises. All exercises were performed with 4 sets. Rest between MJ and SJ sets were 2.5–3 and 1.5–2 min, respectively. All training sessions were supervised by an exercise specialist to ensure safety and adherence to the protocol (Gentil and Bottaro, 2010), and minimum training attendance was set at 80% (Gentil and Bottaro, 2013). During the study period, all participants were engaged in the same specific soccer routine and no other activity was performed outside the study protocol.

**Table 1:** Training programs for the multi-joint (MJ) and single joint (SJ) groups.

**Monday**		**Wednesday**		**Friday**	
**MJ**	**SJ**	**MJ**	**SJ**	**MJ**	**SJ**
Bech press	Peck deck machine	Leg press	Knee extension	Lat pulldown	Pullover
Incline bench press	Incline dumbell fly	Squat	Dumbell lateral raise	Seated row	Rear del fly
Dedlift	Biceps curl	Military press	Abdominal Crunches	Calf Raises	Pulley elbow extension
Abdominal Crunches	Knee flexion	Abdominal Crunches		Abdominal Crunches	Calf Raises
	Abdominal Crunches				Abdominal Crunches

### Statistical analysis

Data is expressed as mean and standard deviation. Unpaired *t*-test was used to compare variables between groups at baseline. Normality of distribution for outcome measures was tested using the Shapiro Wilks test. Paired *t*-tests were used for within group comparisons. Analysis of covariance (ANCOVA) was used to compare post training values between groups, using baseline values as covariates. Significance was set as *p* < 0.05. Data analysis was performed using version 22 of SPSS software package (Statistical Package for Social Sciences, version 22, SPSS Inc., Chicago, Ill).

## Results

The two groups were homogenous at baseline. There were no significant differences between groups for age (23.5 ± 2.7 for SJ and 25.5 ± 3.8 years for MJ) and height (182.1 ± 5.2 for SJ and 185.3 ± 3.6 cm for MJ). The results obtained by SJ and MJ groups are presented in Table 2. There were no changes in body mass in any group. Both groups significantly decreased body fat (6.5 and 11.3% for SJ and MJ, respectively) and increase fat free mass (3.5 and 4.9% for SJ and MJ, respectively), with no difference between groups. Increase in VO_2_max was significant for both groups; however, the 12.5% increase in MJ group was significantly higher than the 5.1% increase seen in the SJ group. Increases in 1 RM in bench press (8.1 and 10.9% for SJ and MJ, respectively), knee extension (12.4 and 18.9% for SJ and MJ, respectively) and squat (8.3 and 13.8% for SJ and MJ, respectively) were significant for both groups. For all these tests, the increases in the MJ group were significantly higher than increases for SJ group (*p* < 0.05).

**Table 2:** Pre and post training values for different variables for single and multi joint groups (mean ± standard deviation).

	**Single joint group**				**Multi joint group**				
	**Pre**	**Post**	**Delta (%)**	***p***	**Pre**	**Post**	**Delta (%)**	***p***	***p*-values for comparison of changes between groups**
Body mass (kg)	80.0 ± 2.8	80.7 ± 3.0	0.8	0.77	81.3 ± 4.0	82.1 ± 3.9	1.1	0.24	0.6
Fat mass (kg)	15.8 ± 1.2	14.8 ± 0.7	−6.5^*^	>0.001	16.6 ± 1.3	14.7 ± 0.8	−11.3^*^	>0.001	0.09
Fat mass (%)	19.7 ± 1.5	18.3 ± 0.8	−7.2^*^	0.01	20.4 ± 1.6	17.9 ± 1.3	−12.2^*^	>0.001	0.31
Fat free mass (kg)	60.3 ± 3.5	62.4 ± 3.3	3.5^*^	0.01	60.9 ± 4.7	64.2 ± 4.9	5.5^*^	>0.001	0.08
VO_2_max (ml/kg.min)	48.7 ± 4.3	51.2 ± 4.8	5.1^*^	0.01	46.5 ± 5.1	52.3 ± 4.8	12.5^*^^.^^#^	>0.001	0.05
Bech press 1 RM (kg)	78.3 ± 9.0	84.7 ± 8.6	8.1^*^	>0.001	80.4 ± 6.8	89.2 ± 7.0	10.9^*^^.^^#^	>0.001	>0.01
Knee extension 1 RM (kg)	80.6 ± 5.7	90.6 ± 4.8	12.4^*^	>0.001	82.2 ± 5.2	97.8 ± 6.0	18.9^*^^.^^#^	>0.001	>0.001
Squat 1 RM (kg)	134.4 ± 7.5	145.6 ± 8.5	8.3^*^	>0.001	139.8 ± 10.4	159.1 ± 14.5	13.8^*^^.^^#^	>0.001	>0.01

* *Significant change whithin groups*.

# *Significant difference between groups*.

## Discussion

The present study aimed to compare the effects of RT performed with MJ or SJ exercises on aerobic capacity, body composition and muscle strength in active males. A novel approach of the present study was equating total work volume which, as far as we know, has not been previously performed. According to our results, both protocols were equally efficient in improving body composition; however, training with MJ exercises provided higher gains in physical performance.

The observed decreases in body fat supports a previous suggestion that RT might be beneficial to promote fat loss (Paoli et al., 2014) and is in agreement with previous studies that reported improvements in body composition in different populations after high intensity RT, even in the absence of caloric restriction (Pratley et al., 1994; Ibañez et al., 2005; Paoli et al., 2010, 2013; Shaw et al., 2016). These positive effects may be due to training intensity, since previous studies showed that high intensity resistance training increases basal metabolic rate and fat oxidation (Melby et al., 1993; Osterberg and Melby, 2000; Paoli et al., 2012).

The molecular events associated with endurance training have been shown to interfere in the in adaptations to resistance training (i.e., decrease gains in muscle strength and hypertrophy); however the interference of resistance training in cardiovascular adaptations is controversial (Baar, 2006). Although increases in cardiorespiratory fitness are not usually associated with RT, recently it has been debated that, when performed at high intensity, RT protocols might provide adequate stimuli to increase cardiorespiratory fitness (Fisher and Steele, 2014). In agreement with this, a review by Steele et al. (2012) suggested that the acute metabolic and molecular responses to high effort RT (i.e., performed to momentary muscular failure) is similar to that of traditional aerobic training. The authors showed evidence that, in the long term, high effort RT produces many physiological adaptations that could help to explain the observed improvements in cardiovascular fitness. These adaptations include increased mitochondrial enzymes, mitochondrial proliferation, conversion of type IIx to type IIa muscle fibers, and vascular remodeling (Steele et al., 2012). Indeed, the 12.5% increase (5.8 ml/kg.min) in VO_2_max seen in the MJ group is similar or even higher than the results usually reported by conventional aerobic training (Milanović et al., 2015). However, it is important to note that the participants were amateur soccer players during the pre-season and specific soccer training might have affected the results. As for the difference between groups, the greater increases seen in the MJ group might be due to more muscles mass involved in the exercises, which would demand a higher oxygen consumption.

According to the present results, training with MJ exercises promoted superior strength gains in all exercises tested, which may be due to the higher neural challenge promoted by MJ exercises. The results of the present study are contrary to the observed by Gentil et al. (2015) who reported similar gains in muscle strength between MJ and SJ as evaluated by isokinetic elbow flexion. The difference between the studies might reside in the differences in protocols and tests used. It has been shown that isokinetic and 1 RM tests are not equivalent and might not be used interchangeably (Gentil et al., 2017b). Therefore, comparing the results of studies that assessed strength through different tests may not be appropriate. The fact that the MJ group was more familiarized with the bench press and squat exercise might explain the differential improvements on these tests, due to movement specificity (Buckner et al., 2017). Notwithstanding, the effects of learning might be questioned since the MJ group also showed higher increases in knee extension.

It is important to note that, in order to equate volume, the SJ group had to train with a higher number of repetitions in the present study. Whilst many studies have found similar strength gains when training at different repetition ranges (Morton et al., 2011; Assunção et al., 2016; Fisher et al., 2017), there are studies reporting higher strength gains in groups that trained with higher loads and low number of repetitions (Campos et al., 2002). According to previous studies, this could be explained by the specificity of the test, since training with a low number of repetitions is closer to what was performed in the 1 RM test (Buckner et al., 2017; Gentil, 2017). Although the literature is equivocal regarding the use of heavy or light loads for RT adaptations (Fisher et al., 2017), we must acknowledge the use of different repetition ranges as a possible limitation of the present study. Whilst comparing the effects of volume-matched SJ and MJ exercise was scientifically necessary, its ecological validity might be questioned, since athletes performing SJ or MJ exercises as part of their RT program will likely perform similar reps and sets for both types of exercise. We must remember that one specific objective of the present study was to compare SJ and MJ protocols with similar volume-load; adopting different repetitions ranges was necessary for that matter. However, future studies are necessary to confirm if the observed differences between the groups are due to the differences in repetitions, and therefore loads used, and not due to SJ or MJ exercises.

In conclusion, the present study suggests that, if one wants to improve body composition, an exercise program composed of either SJ or MJ exercises may be of similar benefit. However, if the purpose is to improve general fitness, performing a resistance training program composed of MJ exercises seems to bring better adaptations than SJ exercises alone.

## Ethics statement

This study was carried out in accordance with the recommendations of Guidelines of the University of Padova Ethics Committee with written informed consent from all subjects. All subjects gave written informed consent in accordance with the Declaration of Helsinki. The protocol was approved by the University of Padova Ethics Committee.

## Author contributions

AP and TM: Designed the work; AP, PG, TM, GM, and AB: Analysis and interpretation of data; AP, PG, and AB: Drafting the work; AP, PG, TM, GM, and AB: Revising critically the work; AP, PG, TM, GM, and AB: Final approval of the version to be published.

### Conflict of interest statement

The authors declare that the research was conducted in the absence of any commercial or financial relationships that could be construed as a potential conflict of interest.
